# *In-Vitro* Fertilization Outcome Predictors in Women With High Baseline Follicle-Stimulating Hormone Levels: Analysis of Over 1000 Cycles From A Tertiary Center

**DOI:** 10.5935/1518-0557.20200088

**Published:** 2021

**Authors:** Gülnaz Sahin, Aysin Akdogan, Murat Hakan Aydın, Mustafa Agah Tekindal, Ege Nazan Tavmergen Göker, Erol Tavmergen

**Affiliations:** 1 Ege University Family Planning and Infertility Treatment and Research Center, Ankara cad., 35100 Bornova, Izmir, Turkey; 2 Deparment of Obstetrics and Gynecology, Ege University Faculty of Medicine, Ankara cad., 35100, Bornova, Izmir, Turkey; 3 Selcuk University, Veterinary Faculty, Department of Biostatistics 42003 Selçuklu, Konya, Turkey

**Keywords:** Aging, assisted reproductive techniques, follicle stimulating hormone, pregnancy rate, live birth

## Abstract

**Objective:**

The present study aimed to evaluate reproductive outcomes and determine the predictors of clinical pregnancy and live birth in women with elevated baseline follicle-stimulating hormone (FSH) levels, who have undergone intracytoplasmic sperm injection (ICSI) treatment.

**Methods:**

This retrospective study included 1011 ICSI cycles of women with high baseline FSH levels (> 10 IU/L), from a tertiary university IVF center between 2010 and 2015. Logistic regression analysis was performed to evaluate the prognostic factors of clinical pregnancy and live birth.

**Results:**

Among the 1011 ICSI cycles, the clinical pregnancy and live birth rates per oocyte retrieval were 19.5% and 14.3%, respectively. The live birth rates were 21.1% and 1.7% in women aged ≤30 years and those aged ≥40 years, respectively. In addition, the live birth rate was 1.47-fold higher in women from whom >3 oocytes were retrieved, compared to those from whom ≤3 oocytes were retrieved (*p*=0.047). Logistic regression analysis indicated that the age categories ≤30y, 36-39y and ≥40y, level of baseline FSH (≥20 IU/L) and the ovarian response (≤3 or >3 oocytes retrieved) were significantly associated with live birth.

**Conclusions:**

Our study indicated that age, baseline FSH level, and ovarian response are independent predictive factors for clinical pregnancy and live birth among women with baseline FSH levels >10 IU/L.

## INTRODUCTION

Prediction regarding assisted reproductive technology (ART) success as a live birth after treatment is an important consideration for the clinician and the infertile couple. Women’s age and ovarian reserve are important markers for ovarian response and; therefore, ART treatment outcomes. Pregnancy rates decrease and miscarriage rates increase with advanced age, and age is one of the most important predictors of *in-vitro* fertilization (IVF) success (Broekmans *et al*., 2006; van Loendersloot *et al*., 2010; Broer *et al.,* 2013; McLernon *et al*., 2016). Both the declining number and the quality of oocytes with aging are associated with decreased success rates in ART cycles.

Ovarian response to stimulation and reproductive potential can be different in women of similar age, according to the individual’s ovarian reserve (ASRM, 2015). Along with women’s age, the association of ovarian reserve and ART outcomes has been the subject of studies (Broer *et al*.,2013; Lukaszuk *et al*., 2014; Ligon *et al*., 2019; Wang *et al*., 2018; Metello *et al*., 2019). Ovarian reserve tests have predictive capacity for ovarian response to gonadotropin stimulation (Jayaprakasan *et al*., 2010; La Marca *et al*., 2010; Broer *et al*.,2013; Nelson *et al*., 2015; Hamdine *et al*., 2015). However, their prediction of pregnancy during ART cycles is limited (Broekmans *et al*.,2006; Broer *et al*., 2013). Anti-Müllerian hormone (AMH), antral follicle count (AFC), and baseline FSH are the most commonly tests used to evaluate ovarian reserve. Although AMH and AFC are more informative biomarkers of ovarian reserve and response (Nelson, 2013; La Marca *et al*., 2016; Tal & Seifer, 2017), baseline FSH level is still one of the commonly used test for daily practices. It is generally accepted that the ovarian reserve is low when the FSH level exceeds 10-12 IU/L (Fang *et al*., 2015). High FSH levels are associated with a low oocyte yield and high cycle cancellation rates (Creus *et al*., 2000; Chuang *et al*., 2003; Abdalla & Thum, 2004; Thum *et al*., 2009; Gingold *et al*., 2015). In a previous meta-analysis, the baseline FSH level, female age, infertility duration, and oocyte number were predictors of IVF outcomes (van Loendersloot *et al*., 2010). Elevated baseline FSH level is one of the used criteria for diagnosis of diminished ovarian reserve (DOR) in clinical practice (Pastore *et al*., 2018). Counseling and management of patients with high baseline FSH levels, as reflecting DOR, needs further consideration.

Assisted reproductive technologies are economical burden and very stressful for couples. Evaluation of the expected pregnancy rate according to the characteristics of the couple is very important, and provides information on the realistic chance of pregnancy. The present study aimed to evaluate reproductive outcomes and determine the predictors of clinical pregnancy and live birth in women with baseline FSH levels >10 IU/L, who have undergone intracytoplasmic sperm injection (ICSI) treatment.

## MATERIALS AND METHODS

This retrospective study included 1011 oocyte retrieval/ICSI cycles of women aged 19-45 years with high baseline FSH (>10 IU/L), and performed in a single tertiary *in vitro* fertilization (IVF) center between 2010-2015. Each patient was included only once. The Institutional Ethics Committee approved the study (No.: 16-2/15). The cycles that used antagonist protocol matched the inclusion criteria. All baseline FSH measurements were performed at the beginning of the ovarian stimulation. Data on clinical variables and controlled ovarian stimulation (COS)/ICSI treatment outcomes were retrieved from the IVF database and the patient medical records. COS was started on day 2 or 3 of the menses, with recombinant FSH or urinary FSH/human menopausal gonadotropin (HMG). When the lead follicle diameter reached 12 mm, the antagonist was added. Human chorionic gonadotropin (hCG) was used for follicular maturation when the lead follicle reached 18 mm. Oocyte aspiration was performed 34-36 hours after hCG administration. All mature oocytes underwent the ICSI procedure 2-4 hours after aspiration. Embryo transfer was performed on day 2-5 of the culture period. According to the directives of the Ministry of Health, a maximum of two embryos were transferred. Progesterone vaginal gel (90 mg/day) was used for luteal phase support. Additionally, a single-dose hCG injection (5000 IU) was administered after day 4 or 5 of the embryo transfer. A visible intrauterine sac on ultrasound 3 weeks after embryo transfer was considered a clinical pregnancy. All pregnancy outcomes were recorded. Miscarriage was defined as a loss of a clinical pregnancy before 24 weeks of gestation. Live birth was defined as the birth of a viable infant after 24 weeks of pregnancy.

The serum baseline FSH levels were quantitatively determined using a chemiluminescent immunometric assay (Access^®^, Beckman Coulter, Inc., Brea, CA, USA) and analyzed using the UniCel^®^ DxI 800 Access^®^ Immunoassay System (Beckman Coulter, Inc.). The range for FSH measurement was 0.2-200 IU/L. Baseline hormone measurements were routinely performed on day 2 or 3 of menses. All measurements were performed at the morning of the start of COS.

To evaluate the influence of age on outcomes, the women were divided into the following groups according to age: group A (≤30 years old), group B (31-35 years old), group C (36-39 years old), and group D (≥40 years old). Additionally, the women were divided into the following groups according to their FSH level: level 1 (10-14.9 IU/L), level 2 (15-20 IU/L), and level 3 (>20 IU/L).

### Statistical analysis

Continuous variables were compared among age groups by variance analysis or the Kruskal-Wallis test, depending on the homogeneity and normality of the variables. For multiple comparisons we used the adjusted Bonferroni (Bonferroni-Dunn) test. Categorical variables were compared using the chi-square test or the Fischer’s exact test where appropriate. Logistic regression analysis was performed to evaluate the predictors of clinical pregnancy and live birth. All statistical analyses were performed using the SPSS software (SPSS ver. 17.0, SPSS Inc., Chicago, IL, USA). Statistical significance was defined as a *p*-value <0.05.

## RESULTS

A total of 1011 oocyte retrieval/ICSI cycles were analyzed. In addition to a decreased ovarian reserve, 7.4% of the cycles were performed for tubal factor infertility and 30.8% were performed for male factor infertility. Table 1 shows the demographics and ICSI outcomes of the study participants. Their mean age was 35.1 ± 4.6 years, their mean FSH level was 14.8±5.1 IU/L, and the mean number of collected oocytes was 4.1±3.7. Fewer than four oocytes were obtained in over half of the cycles (53.9%). Twelve women (1.2%) showed a hyper-response to COS and produced more than 20 oocytes, although their FSH levels were >10 IU/L.

**Table 1:** Demographics and ICSI outcomes of the study participants.

	Overall mean± SD	Overall min-max
**Age (years)**	35 ± 4.65	19-45
**Baseline FSH (IU/L)**	14.8 ± 5.1	10-39.6
**Baseline LH (IU/L)**	6.6 ± 3.3	1-40
**Gonadotropin dose (IU)**	2384 ± 825	900-6650
**No. of oocytes**	4.1 ± 3.7	0-28
**No. of metaphase II oocytes**	3.0 ± 2.8	0-25
**Fertilization rate (%)**	74.5 ± 31.5	0-100
**No. of embryos**	2.5 ± 2.5	0-23
**No. of embryos transferred**	1.3 ± 0.4	1-2
**Clinical pregnancy rate/ opu %, (n)**	19.5	197/1011
**Clinical pregnancy rate/ ET %, (n)**	23.8	197/826
**Live birth rate /opu %, (n)**	14.3	145/1011
**Live birth rate /ET %, (n)**	17.5	145/826

Opu: oocyte pick-up

ET: embryo transfer

Of the 1011 cycles analyzed, 826 (81.7%) involved an embryo transfer procedure. In the remaining 185 cycles (18.3%), embryo transfer could not be performed because of the absence of a mature oocyte (8.2%), fertilization or cleavage failure (7.4%), and other reasons (2.6%). A single embryo transfer was performed in 62.3% of the cycles.

The clinical pregnancy rates were 19.5% (197/1011) per oocyte retrieval, and 23.8% (197/826) per embryo transfer. Biochemical pregnancy was noted in 1.9% (20/1011) of the cycles, and 0.3% (3/1011) had an ectopic pregnancy, which was considered preclinical pregnancy failure. Miscarriage was noted in 5.1% (52/1011) of the cycles and 26.4% (52/197) of clinical pregnancies. The live birth rates were 14.3% (145/1011) per oocyte retrieval and 17.5% (145/826) per embryo transfer cycle.

The ICSI cycle characteristics of the different age groups (groups A, B, C, and D) are presented in Table 2.

**Table 2:** ICSI cycle characteristics of the different age groups.

	Group A ≤30 years n = 180	Group B 31–35 years n = 336	Group C 36–39 years n =379	Group D ≥40 years n = 116	*p*-value
**Baseline FSH (IU/L)**	14.6±5.3	14.1±4.3	14.1±4.3	16.0±5.8^b^	<0.01Ω
**FSH level I (%)** **FSH level II (%)** **FSH level III (%)**	71.1 16.7 12.2	71.7 19.6 8.6	60.4 24.5 15.0	56.9 23.3 19.8	<0.01¥
**Baseline LH (IU/L)**	6.5±2.8	6.5±3.4	6.7±3.6	6.6±2.8	0.9£
**Gonadotropin dose (IU)**	2196±742	2257±773	2555±844^a^^b^	2496±911^a^^b^	0.05Ω
**Peak E2 level (pg/mL)**	1000±884	764±560^a^	654±542^a^	556 ± 476^a^^b^	<0.01£
**No. of oocytes**	6.0±5.1	4.5±3.4^a^	3.4±3.1^a^^b^	2.5±2^a^^b^	0.01Ω
**Poor response (%) (0-3 oocytes)**	37.8	46.4	62.3^a^^b^	73.3^a^^b^^c^	0.001¥
**No. of mII oocytes**	4.2±4	3.3±2.6^a^	2.5±2.4^a^^b^	1.9±1.6^a^^b^	0.01£
**Fertilization rate (%)**	71.7±32.4	75.2±29.1	75±32.5	75.9±34.1	0.9¥
**No. of embryos**	3.5 ± 3.3	2.8 ± 2.3^a^	2.1 ± 2.2^a^^b^	1.5±1.4^a^^b^^c^	0.01£
**No. of embryos transferred**	1.1±0.3	1.2±0.4^a^	1.5±0.5^a^^b^	1.4±0.5^a^^b^	0.01Ω
**Embryo transfer/opu (%)**	83.9	86.9	79.4^b^	70.7^a^^b^^c^	0.001¥
**Clinical pregnancy rate (%)**	23.9 (43/180)	23.5 (79/336)	18.2 (69/379)	5.2(6/116)^a^^b^^c^	0.001¥
**Live birth rate (%)**	21.1 (38/180)	19.3 (65/336)	10.6 (40/379)^a^^b^	1.7 (2/116)^a^^b^^c^	0.001¥
**Miscarriage/clinical preg (%, n)**	11.6 (5/43)	17.7 (14/79)	42.0 (29/69)^a^^b^	66.7 (4/6)^a^^b^	0.001¥

a Different from group A

b Different from group B

c Different from group C

Ω One Way ANOVA

¥ Chi Square Test

£ Kruskal-Wallis Test

Pairwise Comparison with Bonferroni and Bonferroni-Dunn Test

Although the total gonadotropin used was significantly lower in women aged <36 years than in those aged ≥36 years, their peak estradiol level, number of total and metaphase 2 oocytes, and the number of embryos was significantly higher than those who were older. Additionally, an increase in age was significantly associated with a high rate of poor response (0-3 oocytes) (*p*<0.05). Among women aged ≥40 years, the rate of poor response was significantly high (73.3% of the cycles, *p*<0.001) (Table 2). Additionally, there were negative correlations between the number of oocytes with age (r = -0.309, *p*<0.001) and baseline FSH levels (r = -0.313, *p*<0.001).

**Figure 1:**
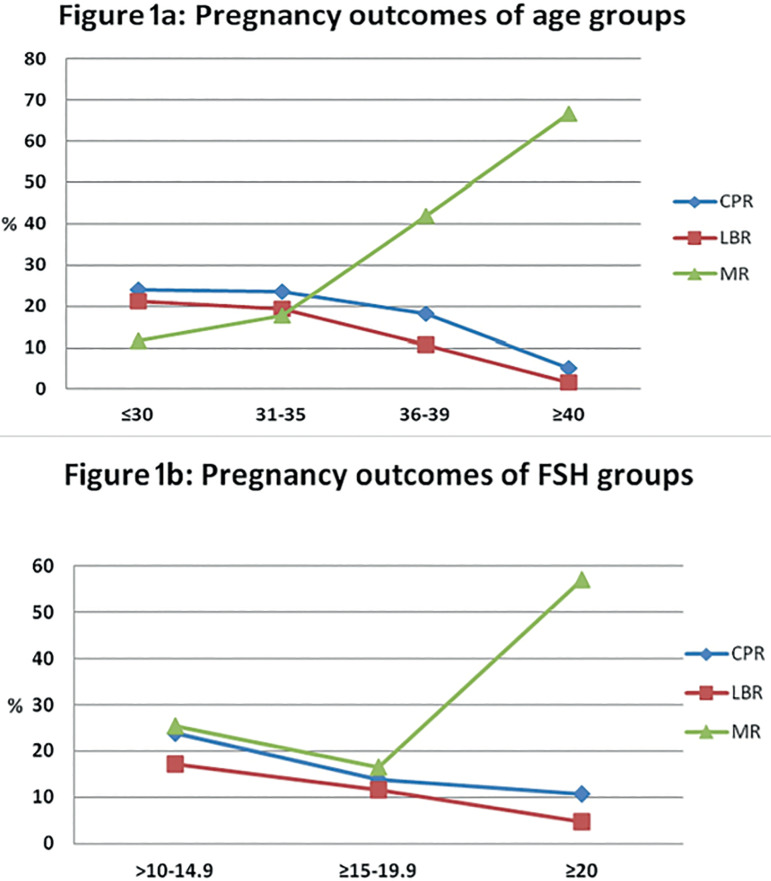
Pregnancy outcomes. 1a. Pregnancy outcomes according to age. 1b. Pregnancy outcomes according to FSH levels.

Among women aged ≤30 years, the clinical pregnancy and live birth rates were 23.9% and 21.1%, respectively. Although the majority of women (89.4%) in this group had a single embryo transfer, the outcomes were acceptable. On the other hand, among women aged ≥40 years, the clinical pregnancy and live birth rates were 5.2% and 1.7%, respectively. The miscarriage rate significantly increased with age (*p*<0.05) and baseline FSH level (Fig 1a, 1b). The miscarriage rates of clinical pregnancies were 42% and 66.7% among women aged 36-39 years and those aged ≥40 years, respectively.

(%) CPR: Clinical pregnancy rate per opu

(%) LBR: Live birth rate per opu (%)

MR: Miscarriage rate per clinical pregnancy

We ran a logistic regression analysis to evaluate the factors related to clinical pregnancy and live birth. The categories of age, level of baseline FSH, ovarian response (≤3 or >3 oocytes retrieved) and number of transferred embryos were applied to the model. Categories of age (≤30, 36-39, ≥40), extreme baseline FSH level (≥20 IU/L) and ovarian response (≤3 or >3 oocytes) were found as independent predictors of live birth (Table 3).

**Table 3:** Evaluation of the predictors of clinical pregnancy and live birth.

	Clinical pregnancy			Live Birth		
	*p* value	OR	95% CI	*p* value	OR	95% CI
**Age ≤30 years**	.008			.001		
**Age 31-35 years**	.773	.936	.595-1.472	.495	.848	.528-1.362
**Age 36–39 years**	.227	.729	.436-1.217	.006	.453	.257-.801
**Age ≥40 years**	.001	.211	.082-.538	.001	.080	.018-.350
**Baseline FSH 10-14.9 IU/L**	.209			.078		
**Baseline FSH 15-20 IU/L**	.128	.705	.449-1.106	.460	.832	.511-1.355
**Baseline FSH ≥20 IU/L**	.040	.698	.373-1.305	.027	.373	.155-.895
**No of oocytes (>3)**	.001	1.846	1.274-2.645	.047	1.474	.978-2.222
**No. of transferred embryos**	.059	1.451	.986-2.136	.282	1.270	.822-1.962

As expected, an increase in age was significantly associated with decreased live birth rates in multivariate analyses. The live birth rate was 2.2-fold lower in women aged 36-39 years than in those aged ≤30 years (*p*=0.006). Conspicuously, the live birth rate was 12.48-fold lower in women aged ≥40 years than in those aged ≤30 years (*p*=0.001). The baseline FSH level was another significant factor for live birth. The live birth rate was 2.68-fold lower in women with baseline FSH levels >20IU/L than in those with FSH levels of 10-14.9 IU/L (*p*=0.02). The number of retrieved oocytes was another significant factor for clinical pregnancy and live birth. The live birth rate was 1.47-fold higher in women from whom >3 oocytes were retrieved than in those from whom ≤3 oocytes were retrieved (*p*= .047).

To find miscarriage predictors, we used logistic regression analysis, and only age was found to be an independent factor for predicting miscarriage.

## DISCUSSION

The present study showed that women with high baseline FSH levels have different cycle outcomes according to age. The pregnancy and live birth rates were higher in younger women than in older women, although all had high baseline FSH levels. In addition to age, the extreme FSH level (>20 IU/ml) and response to stimulation (obtaining ≤3 or >3 oocytes) were independent factors affecting live birth in our large cohort of women with high FSH levels. When the retrieved oocyte number exceeded 3, the clinical pregnancy and live birth chances increased 1.84 and 1.47-fold, respectively, in our cohort. These findings indicate that if a woman responds well to gonadotrophin stimulation, the possibilities of a pregnancy and live birth increase. Thus, efforts to obtain additional oocytes are much valuable in this group of women.

The prediction of pregnancy after IVF is an important issue in reproductive medicine. Several studies have attempted to develop prediction models for IVF outcomes (Nelson & Lawlor 2011; van Loendersloot *et al*.,2013; McLernon *et al*., 2016; Vaegter *et al*., 2017; Leijdekkers *et al*., 2018). Reproductive aging is a well-known cause of poor ovarian reserve, and age is the most established predictor of IVF outcomes (Vaegter *et al*., 2017). Apart from poor ovarian response with aging, studies have found a high rate of chromosomal aberrations that can lead to miscarriage in older women (Bartmann *et al*., 2004; Demko *et al*., 2016; Simon *et al*., 2018). Therefore, the age-related decline in oocyte quality is an important factor in these women.

Consistent with the previous reports, in the present study, women aged ≥40 years with high baseline FSH levels showed a high poor response rate of 73.3% as less than four oocytes were retrieved. Additionally, the live birth probability was 12.4-fold lower in these women than in those aged ≤30 years. Moreover, the rate of miscarriage per clinical pregnancy constantly increased with age; and among women aged ≥40 years; the rate of miscarriage per clinical pregnancy was high at 66%. In our study, only age was found to be significantly associated with miscarriage, and this finding supports the belief that aging is associated with a decline in oocyte quality. Although the miscarriage rate increased as the FSH level increased, this association was not found to be significant in the multivariate analysis.

In some women, fertility declines at an earlier age. Therefore, the ovarian reserve might differ among women of the same age. The term “ovarian reserve” is considered as the number and quality of follicles present in the ovary at “any given time” (Broekmans *et al*., 2006). Although the antral follicle count and anti-Müllerian hormone levels are more informative biomarkers of ovarian response (Nelson, 2013), an elevated early follicular FSH level is considered one of the indicators of a diminished ovarian reserve (DOR) (Chuang *et al*., 2003); and in daily practice, it is one of the commonly used tests for DOR (Buyuk *et al*., 2011). It has been generally accepted that the ovarian reserve is low when the FSH level exceeds 10-12 IU/L (Fang *et al*., 2015), and high FSH levels have been shown to be associated with low oocyte yield (Thum *et al*., 2009; Gingold *et al*., 2015). A previous model for the prediction of ovarian response in antagonist cycles showed that age, antral follicle count, and baseline FSH and LH levels could predict the ovarian response (Broekmans *et al*.,2014). Similarly, in antagonist protocols, the baseline FSH, anti-Müllerian hormone, and antral follicle count were found to be good predictors for ovarian stimulation (Tsakos *et al*.,2014). In addition, nomograms aiming to calculate the starting gonadotropin dose according to age and the FSH, anti-Müllerian hormone, and antral follicle count have already been published (La Marca *et al*., 2012; 2013). Papaleo *et al*. (2016) suggested this nomogram as a useful tool to selecting the gonadotropin dose for stimulation in ART treatments.

van Rooij *et al*. (2003) evaluated the influence of age and FSH level on IVF outcomes. They found that aging mainly affects the implantation and pregnancy rates owing to poor oocyte quality and that elevated FSH levels are associated with cycle cancellation owing to the poor response to gonadotropins. They stated that the implantation and pregnancy rates might be reasonable if a young woman with high FSH levels produce oocytes. Chuang *et al*. (2003) suggested that age, but not the FSH level is independently associated with pregnancy rates, and both a high FSH level and aging are significantly associated with the numbers of oocytes collected, fertilized oocytes, and transferred embryos. These results are consistent with the conclusion that age is a better predictor of oocyte quality, and baseline FSH level is a better predictor of oocyte number (Toner, 2003).

According to our study results, both high FSH levels and aging are significantly associated with reduced numbers of oocytes. Consistent with the findings of previous studies, our study showed that in young women (aged ≤30 years) with poor ovarian reserve, clinical pregnancy and live birth rates were acceptable at 23.9% and 21.1%, respectively, although the majority of the women had a single embryo transfer. On the other hand, in the present study, not only age but also the extreme baseline FSH level (>20 IU/L) was found to be a significantly independent predictor of pregnancy and live birth. This observation was consistent with the finding of a previous meta-analysis that included pooled data from five studies, which showed that a high baseline FSH level is associated with low pregnancy rates and that FSH is one of the significant predictive factor for pregnancy outcome (van Loendersloot *et al*., 2010).

The outcomes of low-prognosis patients have been of a special interest in the field of ART. Chang *et al*.(2018), reported lower clinical pregnancy rates with higher miscarriages in older women with diminished ovarian reserve (defined by elevated FSH ≥10 IU/L, AMH level of <1.1ng/ml and AFC<6 ), than in younger women with diminished ovarian reserve. Levin *et al*. (2015) evaluated the IVF outcome in women with baseline FSH levels of 10.0-11.9 IU/L, which were considered as “borderline FSH levels.” The authors reported that the number of embryos was the only predictor of clinical pregnancy. Huang *et al*. (2015) evaluated the predictive factors of IVF outcomes in 58 women with high baseline FSH levels (≥12 IU/L). They found that ovarian stimulation parameters (the number of mature follicles on trigger day, number of oocytes, and developed embryos) are predictive factors for the IVF outcome in those selected group. Kushnir *et al*. (2018), evaluated IVF outcomes of 291 women with high baseline FSH levels (>20 IU/L). The highest live birth rates are from women <35 years at (17.2%), and the lowest rates from women>42 years (1.9%).

The most important endpoint is the live birth rate of ART treatments. We attempted to analyze the live birth rates and predictive factors for live birth in our large data set of women with baseline FSH levels >10 IU/L. The strength of this study is that it included more than 1000 oocyte retrieval cycles from a single tertiary center. The limitation is the retrospective nature of the study. In addition, we collected the data from patients who underwent oocyte retrieval; therefore, the prior cancellation before the oocyte retrieval procedure was not included in this study.

In conclusion, age, baseline FSH level, and the ovarian response as; ≤3 or >3 oocytes retrieved, are significant predictors of clinical pregnancy and live birth among women with high baseline FSH level. These findings can be used to appropriately counsel women undergoing ART in order to ensure that these women have realistic expectations.
